# Induction of auxin biosynthesis and *WOX5* repression mediate changes in root development in Arabidopsis exposed to chitosan

**DOI:** 10.1038/s41598-017-16874-5

**Published:** 2017-12-01

**Authors:** Federico Lopez-Moya, Nuria Escudero, Ernesto A. Zavala-Gonzalez, David Esteve-Bruna, Miguel A. Blázquez, David Alabadí, Luis V. Lopez-Llorca

**Affiliations:** 1 Laboratory of Plant Pathology, Multidisciplinary Institute for Environment Studies (MIES) Ramón Margalef, Department of Marine Sciences and Applied Biology, University of Alicante, Alicante, Spain; 2 Departament d’Enginyeria Agroalimentària i Biotecnologia, Universitat Politècnica de Catalunya, Barcelona, Spain; 3 Atlántica Agrícola Company SA. Villena, Alicante, Spain; 4 Instituto de Biología Molecular y Celular de Plantas, Consejo Superior de Investigaciones Científicas-Universidad Politécnica de Valencia, Valencia, Spain

## Abstract

Chitosan is a natural polymer with applications in agriculture, which causes plasma membrane permeabilisation and induction of intracellular reactive oxygen species (ROS) in plants. Chitosan has been mostly applied in the phylloplane to control plant diseases and to enhance plant defences, but has also been considered for controlling root pests. However, the effect of chitosan on roots is virtually unknown. In this work, we show that chitosan interfered with auxin homeostasis in Arabidopsis roots, promoting a 2–3 fold accumulation of indole acetic acid (IAA). We observed chitosan dose-dependent alterations of auxin synthesis, transport and signalling in Arabidopsis roots. As a consequence, high doses of chitosan reduce *WOX5* expression in the root apical meristem and arrest root growth. Chitosan also propitiates accumulation of salicylic (SA) and jasmonic (JA) acids in Arabidopsis roots by induction of genes involved in their biosynthesis and signalling. In addition, high-dose chitosan irrigation of tomato and barley plants also arrests root development. Tomato root apices treated with chitosan showed isodiametric cells respect to rectangular cells in the controls. We found that chitosan causes strong alterations in root cell morphology. Our results highlight the importance of considering chitosan dose during agronomical applications to the rhizosphere.

## Introduction

Soil-borne pathogens cause some of the most serious diseases of cultivated crops and pose a serious threat to global food security^1^. Infections caused by soil borne organisms such as nematodes, fungi or bacteria can result in crop losses of more than $120 billion dollars per year in the USA only^2^. These losses together with the restriction in the use of fungicides, bactericides and nematicides justify the need to study and develop more sustainable methods of control. Chitosan has been used in agriculture to control plant damage by viruses and viroids, bacteria, fungi, nematodes and other pests^3^. Chitosan is a highly deacetylated form of chitin, with numerous applications in agriculture^4^. Chitosan is biodegradable, friendly to the environment and non-toxic to mammals and humans in particular^5,6^.

Chitosan is known to cause important physiological changes in plants^7^ such as growth stimulation of *Eustoma grandiflorum* seedlings (Raf.) Shinn^8^ and *Robusta coffee* (L.)^9^ or enhancing seed germination of *Dendrobium* spp.^10^. In addition, chitosan also promotes tomato^11^ and orchid production^12^, reduces flowering time of *Dendrobium* spp.^13^ and stimulates plant tissue differentiation^14^. Chitosan has been described as enhancer of photosynthetic rate in plants^15^ and modulator of their nutritional status. Chitosan acts as elicitor^16^ of plant secondary metabolites such as alkaloids^17^, withanolides^18^ and lignin^19^.

However, chitosan has mainly been applied to the phylloplane for controlling pests and diseases or modifying plant growth and defences. The effect of its aerial application has been also described as a stress-like response, which translates into the priming of plant defences^20–22^. Chitosan induces oxidative stress in tomato plants^23^ as well as in fungi^6,24^. Response to chitosan in plants is mediated by stomatal closure, which is independent of endogenous abscisic acid or jasmonates^25^. The manipulation of the plant immune system by using chitosan has recently been reviewed^26^. However, less is known about the effect on root development when chitosan is applied in the rhizosphere. Baque *et al*.^27^ found that chitosan applied to adventitious root cultures enhances secondary metabolite production and decreases root growth on *Morindia* plants. On the contrary, Khalil & Badawy (2012)^28^ found promotion of root growth by chitosan but applied only once and in plants inoculated with the root-knot nematode *Meloidogyne incognita*. Still the direct effect of chitosan on plant root at physiological and cellular levels is not known.

In this study we investigate the effect of chitosan on root development trying to establish the basis for its application in agriculture. We use the model plant *Arabidopsis thaliana* (Arabidopsis) as well as barley and tomato plants which are profitable crops and important for food security. We have investigated the activity of chitosan on plant growth and physiology, and in particular the effect of chitosan on root cell architecture and organisation. We study the production of plant hormones in roots treated with chitosan. We have also determined the activity of chitosan on gene expression regulating auxin, jasmonic and salicylic acids biosynthesis, signalling and regulation. In addition, we have also analysed how chitosan acts on quiescent centre (QC) organisation in Arabidopsis root meristem. In this paper, we have also applied chitosan in the irrigation system of tomato and barley plants. We have determined the effect of chitosan on plant growth and apical root meristem cell dynamics. This work provides a better understanding of the mode of action of chitosan on root development, focusing on hormone homeostasis.

## Results

### Chitosan reduces root growth and development in *Arabidopsis*

**Figure 1:**
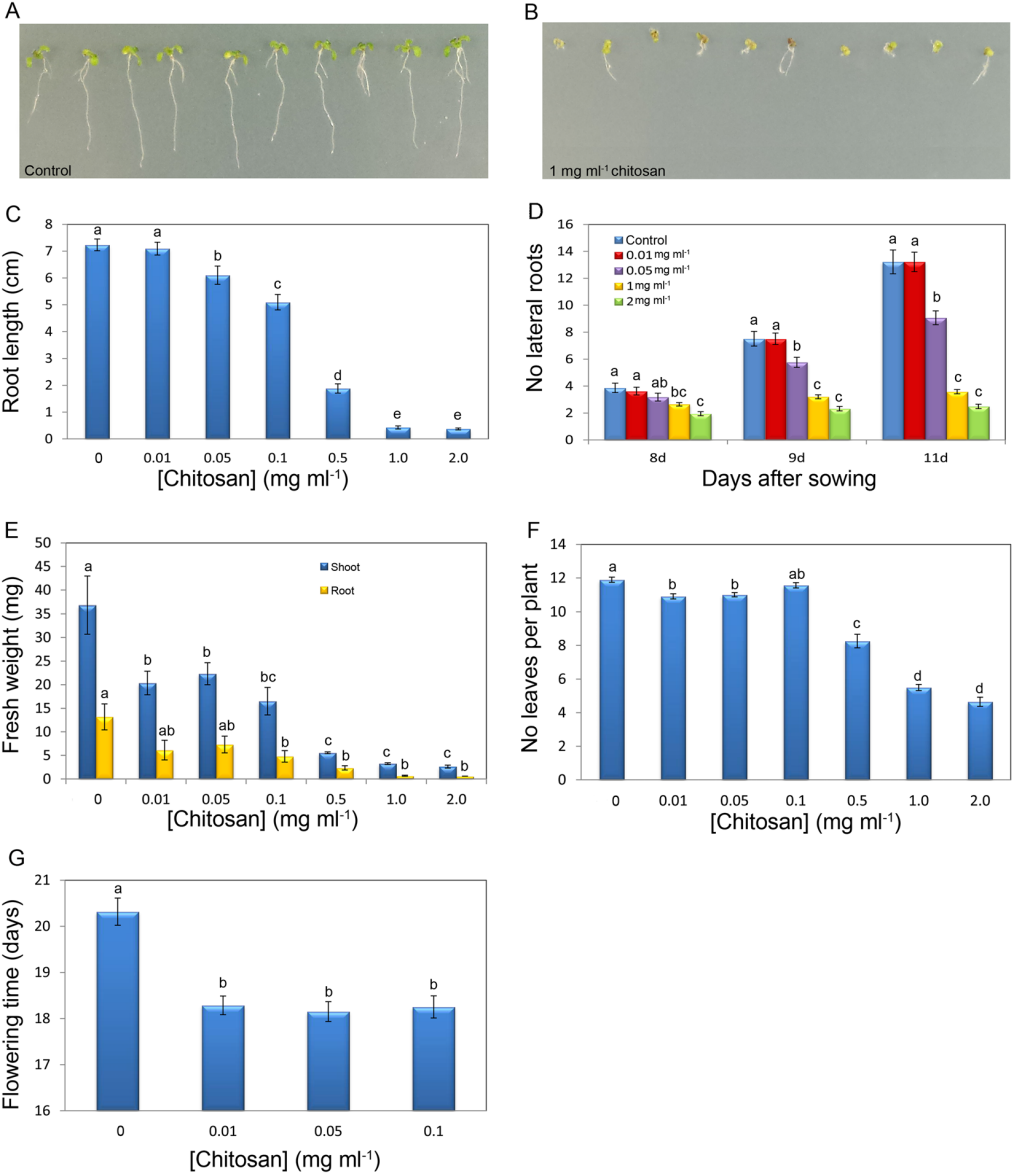
Chitosan alters Arabidopsis growth and development. (**A**) Untreated (control) plants (**B**) Plants treated with 1 mg ml^−1^ chitosan. (**C**) Effect of chitosan on root length. (**D**) Chitosan affecting secondary root formation. (**E**) Effect of chitosan on shoot and root weight. (**F**) Activity of chitosan on leaf production. (**G**) Evaluation of the activity of chitosan on flowering time.

To investigate the possible effects on root growth of chitosan applied to the rhizosphere, we examined the phenotype of Arabidopsis plants (Col-0) treated with increasing doses of chitosan (Fig. 1A and B). After 21d, low doses of chitosan (0.01–0.1 mg ml^−1^) have little impact on Arabidopsis growth, however higher chitosan concentrations (>0.5 mg ml^−1^) severely arrested Arabidopsis root development (Fig. 1C). Plants in contact with 2 mg ml^−1^ chitosan for 11 days, displayed 80% reduction (p-value < 0.05) in the number of secondary roots respect to controls (Fig. 1D). However, lower doses of chitosan (0.05 and 0.1 mg ml^−1^) only caused a slight reduction of root biomass. Arabidopsis roots exposed to 1 mg ml^−1^ chitosan solution for 2 h showed an accumulation of reactive oxygen species (ROS) (Fig. S1). This suggests chitosan causes a systemic stress response.

Chitosan (0.5 mg ml^−1^ and upwards) also arrested shoot development (Fig. 1E). The number of leaves per plant also decreased significantly (p < 0.05) in plants grown with 0.5 mg ml^−1^ chitosan upwards (Fig. 1F). Besides, low chitosan doses (0.01–0.1 mg ml^−1^) significantly reduce flowering (bolting) time (Fig. 1G). Indicating that low chitosan doses cause slight stress which is translated in to flowering induction. However, plants treated with high chitosan doses (>0.5 mg ml^−1^), exposed to high stress, did not flower after 30d.

### Chitosan modifies root growth repressing *WOX5*

**Figure 2:**
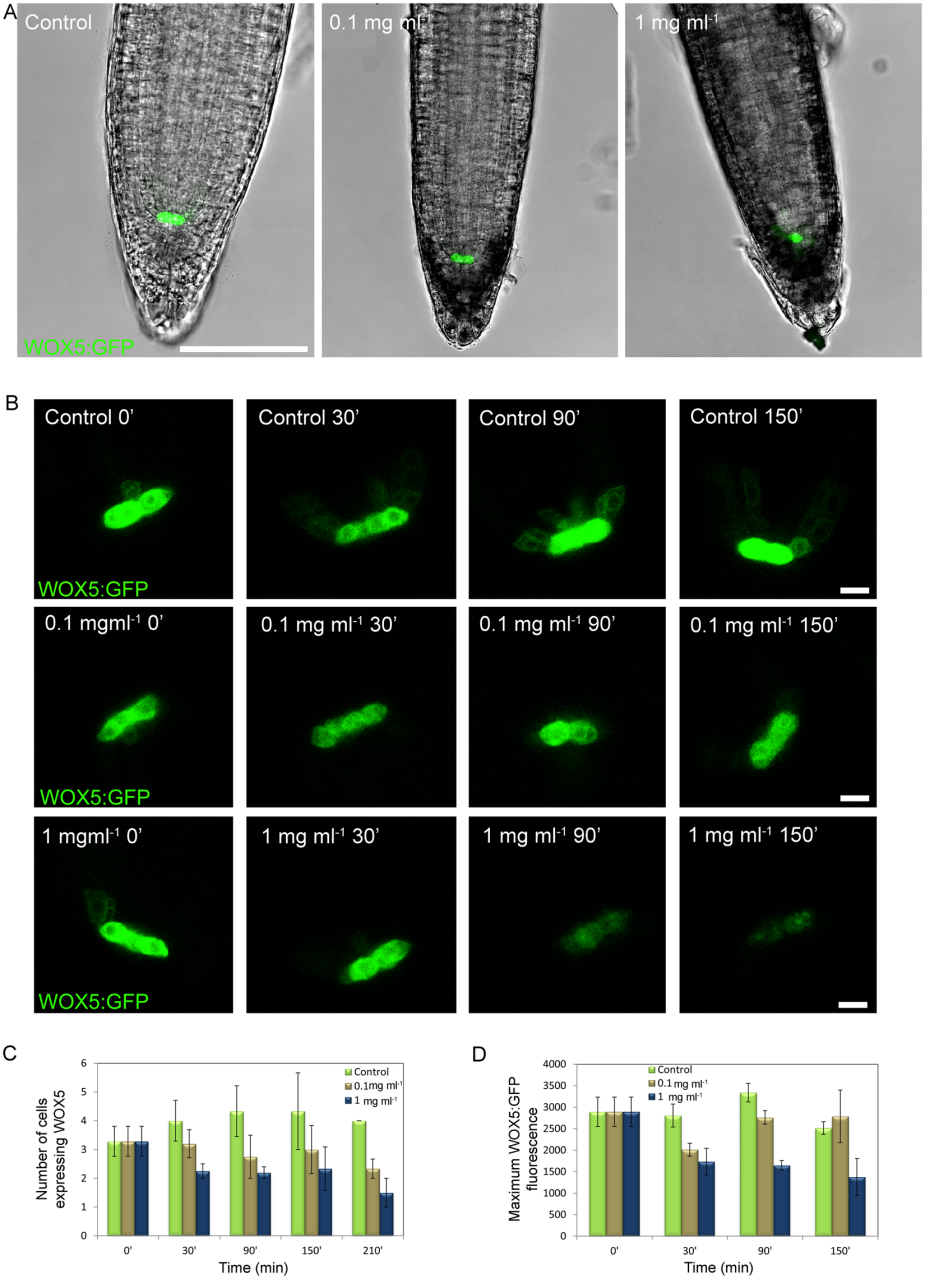
Altered root development by chitosan correlates with the repression of the quiescent centre gene *WOX5*. (**A**) Tips from 5d-old-roots after 150 min in contact with MS medium (control), 0.1 and 1 mg ml^−1^ chitosan. (Scale bar 75 µm). (**B**) Time-course expression of *WOX5* in roots exposed to MS medium amended with chitosan. Untreated controls were also included. (Scale bar 5 µm). (**C**) Number of cells expressing WOX5:GFP. (**D**) Maximum fluorescence emission from cells expressing WOX5:GFP.

Chitosan altered root physiology and cellular activity. After 30 min with chitosan, roots did not display visual changes. However, after 90 min exposure to 1 mg ml^−1^ chitosan roots became dark and displayed auto-fluorescence in the root cap and epidermal cells when excited at 580–620 nm (Fig. 2A and Fig. S2). After 150 min, roots exposed to 0.1 mg ml^−1^ chitosan displayed these dark/auto-fluorescent compounds (likely phenols) only in root tips. Whereas at this time point (150 min) larger chitosan dose (1 mg ml^−1^) caused widespread accumulation of these compounds in the roots (Fig. 2A).

Morphological changes observed in roots treated with chitosan led us to characterize the activity of the root meristematic quiescent centre (QC) (Fig. 2 and Fig. S2). Five-day-old plants placed in chitosan solutions displayed changes in the root apex that correlated with miss-expression of the *WUSCHEL RELATED HOMEOBOX 5* (*WOX5*) gene. *WOX5* encodes a transcription factor key to stem cell maintenance in the QC located using WOX5-GFP^29^ (Fig. 2A). In untreated roots, WOX5-GFP expression was observed in the two stem cells of the QC and to a lower extent in the neighbouring cells. Chitosan (0.1–1 mg ml^−1^) reduced both the expression level in these cells (Fig. 2B) and also the number of root cells expressing *WOX5* (Fig. 2C). In roots exposed to high doses of chitosan (1 mg ml^−1^) expression of *WOX5* in cells of the QC was clearly reduced after 90 min, and by 150 min expression was almost lost (Fig. 2B). The reduction in *WOX5* expression was confirmed by fluorescence quantification in primary (Fig. 2D) and secondary roots (Fig. S3).

In view of the activity of chitosan on *WOX5* expression and localisation, we also evaluated the activity of this polymer on *ACTIVE QUIESCENT CENTRE* (*AQC1*) gene expression (Fig. S4). We demonstrated that low doses of chitosan (0.1 mg ml^−1^) induced *AQC1* gene expression more than 5-fold. However, high chitosan dose (1 mg ml^−1^) repressed this gene as happened with *WOX5* in roots. This result is in agreement with the reduction of root growth in plants exposed to high chitosan doses.

### Chitosan induces changes in hormone levels in Arabidopsis roots

In view of the changes in *WOX5* and *AQC1* gene expression caused by chitosan and since the expression of these genes is regulated at least by auxin^30,31^, we evaluated the hormonal status in response to the polymer. Roots from Arabidopsis plants grown 3d on MS solid medium amended with 1 mg ml^−1^ chitosan increased 3.6-fold (p < 0.05) auxin (IAA) content respect to controls. Chitosan also significantly increased (2–3 fold; p < 0.05) salicylic acid (SA) and jasmonic acid (JA) compared with plants not exposed to this polymer (Table 1). However, levels of abscisic acid (ABA) were slightly reduced in roots exposed to chitosan. We also observed that the levels of the active gibberellic acid (GA4) did not change in chitosan-treated roots.

**Table 1:** Activity of chitosan on plant hormones in Arabidopsis roots. Chitosan induces accumulation of auxins measured by accumulation of IAA. Chitosan also propitiates accumulation of JA and SA hormones significantly. Asterisks indicate significant differences *(p < 0.05) respect to control (no chitosan).

		Control	1 mg ml^−1^ chitosan
		[Hormone] ng g^−1^ root	[Hormone] ng g^−1^ root
Hormones	GA4	0.08 ± 0.01	0.04 ± 0.001
	ABA	2.39 ± 0.14	0.49 ± 0.07*
	JA	13.88 ± 1.41	35.14 ± 2.27*
	SA	28.82 ± 3.16	79.88 ± 4.98*
	IAA	46.56 ± 5.30	167 ± 11.77*

GA4: gibberellic acid; ABA: abscisic acid; JA: jasmonic acid; SA: salicylic acid; IAA: indole acetic acid.

### Chitosan induces tryptophan-dependent pathway for IAA biosynthesis

**Figure 3:**
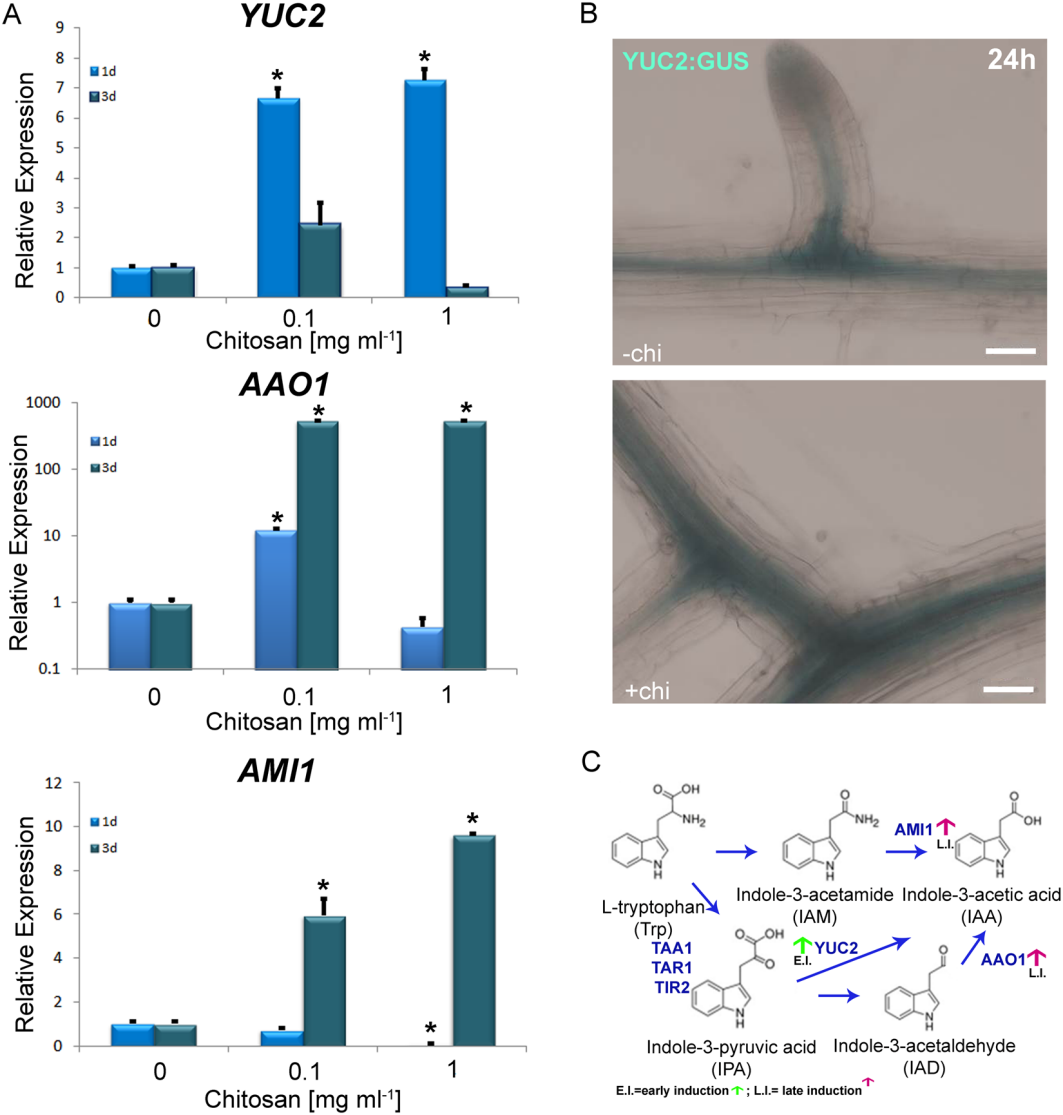
Chitosan induces the Trp-dependent pathway for auxin synthesis. (**A**) Exposition of Arabidopsis plants to chitosan for 1d induces relative expression of *YUC2*. *AAO1*is overexpressed after 3d in contact with 1 mg ml^−1^. However, *AMI1* is repressed after 1d in contact with chitosan. Higher doses of chitosan (1 mg ml^−1^) allows overexpression of *AMI1* after 3d in contact with chitosan. Besides, low doses of chitosan (0.1 mg ml^−1^) induces Trp-dependent pathway for auxin synthesis. Arabidopsis plants exposed for 1d to low chitosan doses propitiates overexpression of *YUC2*. However, Arabidopsis plants exposed for 3d to low chitosan doses propitiates overexpression of *AAO1* and *AMI1*. Asterisks indicate significant differences *(p < 0.05) respect to control (**B**) Histochemical analysis shows chitosan induces YUC2:GUS transgene expression in Arabidopsis roots. (Scale bar 50 µm). (**C**) Simplified view of IAA and its modulation by chitosan.

To determine if the increase in auxin levels is due to transcriptional changes in auxin biosynthetic genes, we tested the expression of genes that participate in the Trp-dependent pathway that is the major auxin biosynthetic pathway in Arabidopsis. Chitosan induced IAA accumulation in Arabidopsis roots via L-tryptophan (Trp) dependent pathway (Fig. 3). Chitosan (1 mg ml^−1^) in MS solid medium induced *YUCCA2* (*YUC2*) gene expression more than 8 fold in Arabidopsis plants exposed to this polymer for 1d (Fig. 3A). We confirmed this result using YUC2:GUS plants exposed to 1 mg ml^−1^ chitosan solutions for 24 h. We showed *YUC2* overexpression in the vascular tissue and in zones of secondary root differentiation (Fig. 3B). However, chitosan repressed this gene in plants exposed 3d to chitosan. Besides, transcriptional analysis by qRT-PCR also showed a large (ca. 100-fold) overexpression of *ALDEHIDE OXIDASE1* (*AAO1* encoding indole-3-acetaldeyde oxidase) in plants grown for 3d on MS plates amended with 1 mg ml^−1^ chitosan (Fig. 3A). Likewise, *AMIDASE1* (*AMI1* encoding indole-3-acetamide hydrolase) is overexpressed more than 9-fold in plants exposed for 3d to 1 mg ml^−1^ chitosan. These results suggest that late overexpression of *AAO1* and *AMI1* in response to chitosan induced auxin accumulation in roots. We also showed the same trend in the gene expression of those genes related with IAA synthesis at low chitosan doses (0.1 mg ml^−1^) (Fig. 3A). Chitosan induced *YUC2* in plants exposed for 1d to 0.1 mg ml^−1^ chitosan and *AAO1* and *AMI1* in plants exposed for 3d to the same chitosan concentration. In conclusion, exposure of Arabidopsis plants to chitosan triggered IAA synthesis by a tryptophan-dependent pathway which results in an accumulation of IAA in roots (Fig. 3C).

### Chitosan alters IAA transport and signalling

**Figure 4:**
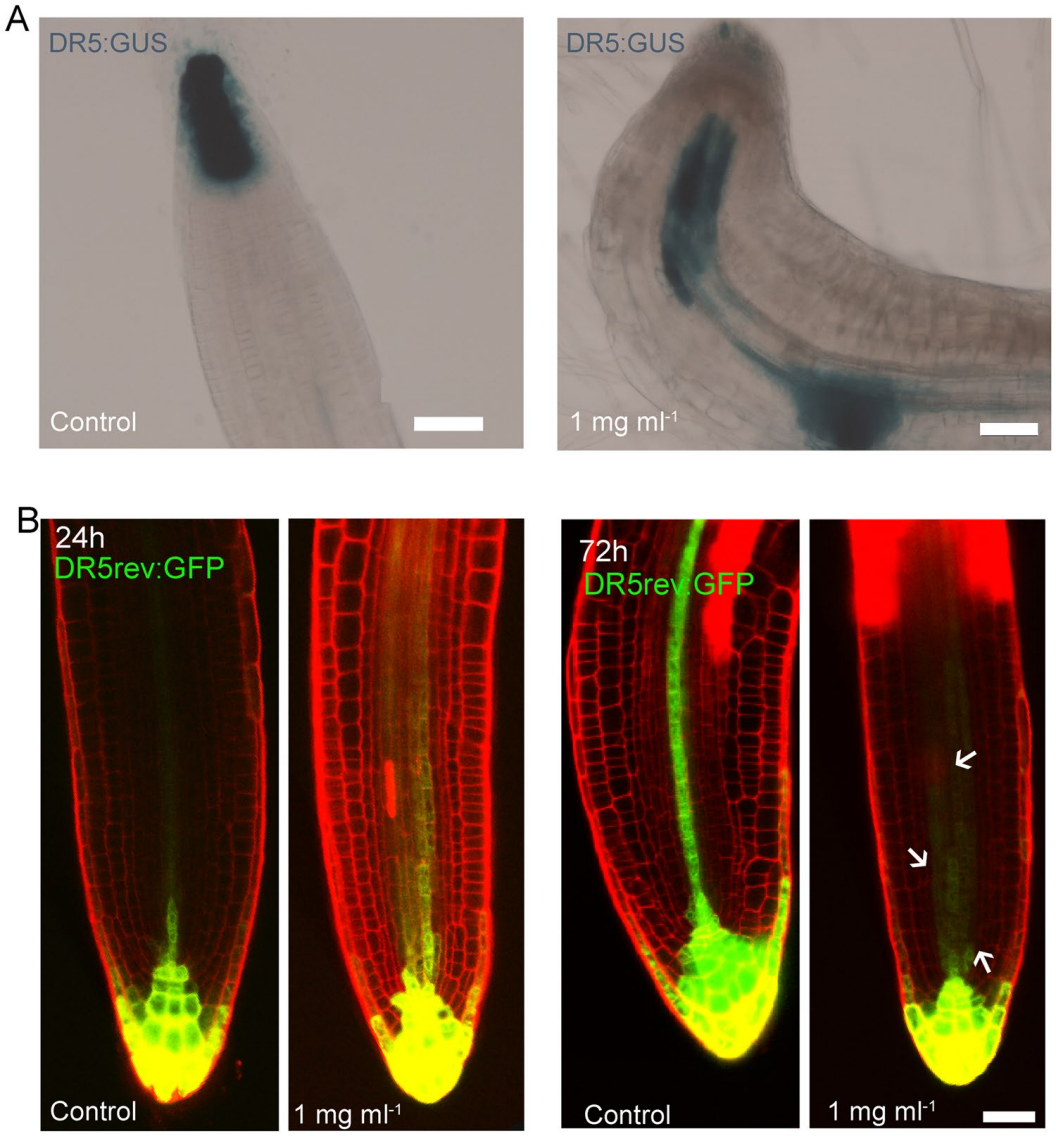
Chitosan causes auxin accumulation in Arabidopsis roots. (**A**) Two hours exposition to liquid chitosan provoke changes in DR5:GUS localisation in primary roots exposed this polymer. Untreated plants shows DR5:GUS expression located in the meristematic zone and stem cell niche. Plants exposed to chitosan showed accumulation of DR5:GUS expression in the transition zone and meristematic zone. (Scale bar 50 µm). (**B**) Chitosan causes mislocalisation of DR5rev:GFP in secondary roots. DR5rev:GFP localise in the meristematic zone and vascular tissue. Arrows indicate expression of *DR5* in the meristematic zone. (Scale bar 50 µm).

**Figure 5:**
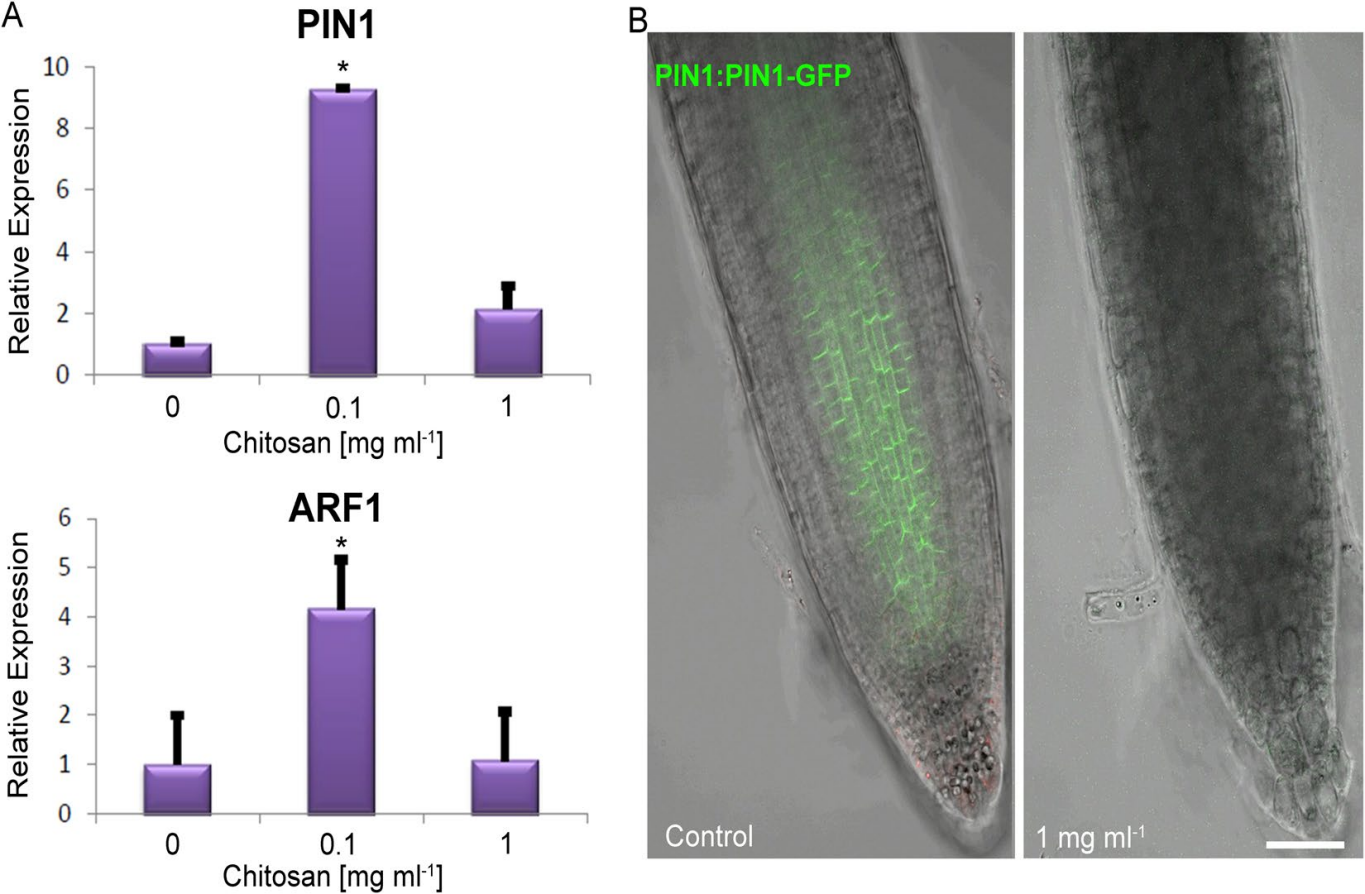
Chitosan modify auxin transport and auxin signalling gene expression in Arabidopsis roots. (**A**) Relative expression of *PIN1* and *ARF1* at low chitosan doses (0.1 mg ml^−1^). High chitosan doses (1 mg ml^−1^) repress expression of both genes. Asterisks indicate significant differences *(p < 0.05) respect to control. (**B**) Chitosan (1 mg ml^−1^) abolishes PIN1-GFP fusion protein visualization in Arabidopsis roots in comparison with untreated Arabidopsis roots. (Scale bar 10 µm).

In agreement with the increase in IAA levels, a 2 h treatment with chitosan (1 mg ml^−1^) enhanced auxin signaling in Arabidopsis primary roots as detected by the DR5:GUS reporter (Fig. 4A). Interestingly, the activity of the reporter extended to the newly emerged secondary roots (Fig. 4B). Despite the fact that this reporter is transcriptional and does not respond directly to auxin levels, it is reasonable to think that the increase in its activity is due, at least partly, to chitosan-induced accumulation of IAA. We also showed that *AUXIN RESPONSE FACTOR1* (*ARF1*), known to repress gene expression in response to auxin^32^, was induced ca. 5-fold at low chitosan dose (0.1 mg ml^−1^) (Fig. 5A), suggesting it is part of the signalling pathway triggered by chitosan. A higher dose of this polymer (1 mg ml^−1^), however, caused no changes in its expression.

We next tested if chitosan was also acting on the transport of auxin. Low doses of chitosan (0.1 mg ml^−1^) induced expression of the auxin efflux carrier gene *PIN1* (Fig. 5A). On the contrary, higher chitosan dose (1 mg ml^−1^) abolished PIN1-GFP signal in Arabidopsis roots (Fig. 5B), suggesting it could potentially affect auxin transport propitiating auxin accumulation in roots. In summary, chitosan can impair root growth and morphogenesis modifying auxin synthesis and delivery.

### Chitosan induces the expression of SA- and JA-related genes

**Figure 6:**
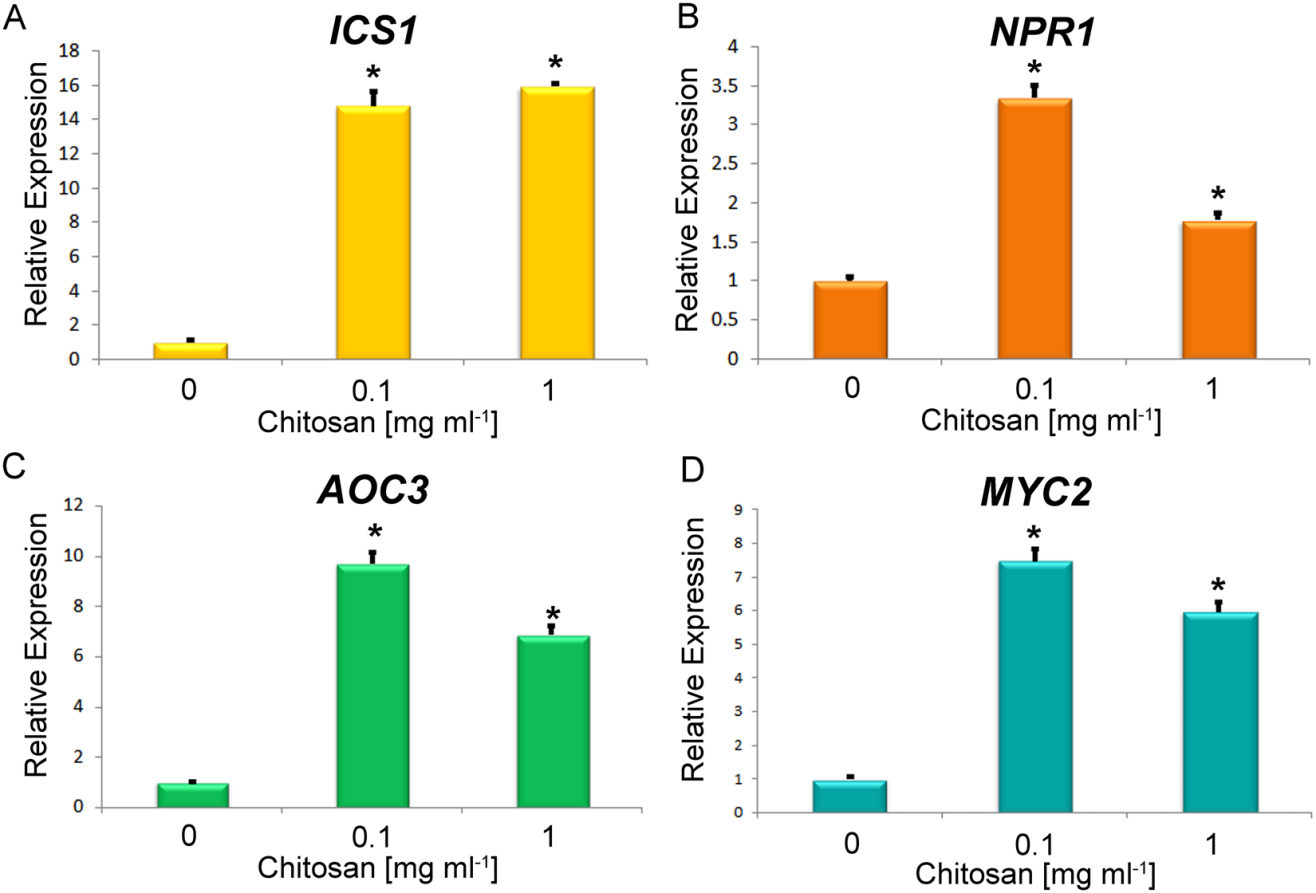
Chitosan induces expression of genes involved in SA and JA biosynthetic and signalling pathways. (**A**) Chitosan significantly (p < 0.05) overexpress SA biosynthetic genes isochorismate synthase 1 (*ICS1*) under low (0.1 mg ml^−1^) and high (1 mg ml^−1^) chitosan doses in Arabidopsis plants (Col-0). (**B**) Chitosan doses (0.1 and 1 mg ml^−1^) induce *NPR1* gene related with SA signalling in Arabidopsis plants. Chitosan also significantly (p < 0.05) induces expression of JA related genes (**C**) *AOC3* (JA biosynthesis) and (**D**) *MYC2* (JA signalling) respect to untreated controls.

Similar to the effect on auxin-related genes, chitosan induced the expression of genes involved in the synthesis and perception of SA and JA (Figs 6 and S5). This supports the increase in JA and SA found in roots of Arabidopsis plants exposed to chitosan (Table 1). More specifically, the expression of genes involved in the isochorismate pathway (*ICS1* and *ICS2*) for SA biosynthesis was induced by chitosan between 6 and 15-fold (Figs 6A and S5A), in agreement with the larger accumulation of SA (Table 1). Chitosan induction (ca. 3.5–1.5 fold) of *NON-INDUCIBLE IMMUNITY PROTEIN* (*NPR1*) gene, required for normal systemic acquired resistance (SAR), also supports a possible enhancement of SA mediated signalling in Arabidopsis exposed to chitosan (Fig. 6B). With respect to JA biosynthesis, *ALLENE OXIDE CYCLASE3* (*AOC3*) was induced 9-fold with low chitosan dose (0.1 mg ml^−1^) (Fig. 6C). The same trend was also found with higher doses of chitosan (1 mg ml^−1^). *LIPOXIGENASE3* (*LOX3*) was only slightly induced by 1 mg ml^−1^chitosan (Fig. S5B). Likewise, *CYTOCHROME P450 94B1* (*CYP94B*) which mediates jasmonoyl-isoleucine hormone oxidation in the last stages of the biosynthesis of the active hormone was also induced (ca. 5-fold) by chitosan (Fig. S5C). An equivalent induction (6–7 fold) was found for *MYC2*, encoding a JA-dependent transcription factor (Fig. 6D). In summary, induction of SA and JA by chitosan could mediate balancing on plant immunity.

### Hormone involvement on root growth defects caused by chitosan

To evaluate if only the changes in hormone levels could explain the reduction of root growth caused by chitosan, wild-type seedlings were grown in the presence of IAA, SA and JA levels equivalent to the concentrations found after chitosan treatment (Table 1). When IAA, SA and JA were applied together, root length was significantly reduced respect to control (Fig. S6A). This reduction was lower than that found in chitosan treated plants. However, the three hormones combined increased root weight (Fig. S6B). This suggests hormone homeostasis of IAA, JA and SA together with other factors such as induction of ROS is involved in the growth reduction caused by chitosan.

We evaluated the role of hormone perception on root growth reduction by chitosan. For this purpose, we tested Arabidopsis mutants with reduced sensitivity to either SA or JA (SA: *npr1–1*; JA: *coi1–40* and *myc2/3/4* triple mutant). After 15d on MS medium amended with chitosan (0.1 mg ml^−1^) JA insensitive mutants (*coi1–40* and *myc2/3/4*) had larger (p < 0.05) root biomass (ca. 2-fold) than these mutants growing on MS only (Table 2). The same phenotype was shown in Col-0 plants amended with IAA and SA (with lack in JA) (Fig. S5). These results suggest that JA could play a relevant role on shortening root by chitosan. Conversely, the mutant with reduced sensitivity to SA (*npr1–1*) did not show significant differences on plant biomass. However, *npr1-1* plants showed a significant rise in number of leaves per plant (ca. 10%; Table 2). On the contrary, higher chitosan dose (1 mg ml^−1^) severely reduced growth of both JA and SA mutants.

**Table 2:** Effect of chitosan on Arabidopsis mutants with reduced JA/SA sensitivity.

Chitosan (mg ml^−1^):	MRL (cm)			RFW (mg)			SFW (mg)			No leaves		
	0	0.1	1	0	0.1	1	0	0.1	1	0	0.1	1
**Col-0**	8.56 ± 0.28^a^	8.96 ± 0.34^a^	1.12 ± 0.06^b^	6.45 ± 0.35^a^	6.74 ± 0.29^a^	0.2 ± 0.02^b^	20.18 ± 0.62^a^	20.48 ± 0.58^a^	1.16 ± 0.09^b^	9.68 ± 0.19^a^	10.13 ± 0.18^a^	3.67 ± 0.19^b^
***npr1–1*** (SA insensitive)	7.08 ± 0.21^a^	7.39 ± 0.45^a^	0.59 ± 0.04^b^	2.15 ± 0.12^a^	3.64 ± 0.27^a^	0.12 ± 0.02^b^	10.7 ± 0.24^a^	13.9 ± 0.49^a^	1.67 ± 0.03^b^	6.53 ± 0.24^a^	**7**.**4 ± 0**.**16** ^**b**^	4.06 ± 0.12^c^
***coi1–40*** (JA insensitive)	8.19 ± 0.38^a^	8.09 ± 0.59^a^	0.71 ± 0.07^b^	3.18 ± 0.25^a^	**5**.**77 ± 0**.**33** ^**b**^	0.15 ± 0.01^c^	12.28 ± 0.63^a^	14.97 ± 0.48^a^	1.47 ± 0.04^b^	8.67 ± 0.25^a^	8.67 ± 0.27^a^	4.34 ± 0.16^b^
***myc2/3/4*** (JA insensitive)	7.63 ± 0.26^a^	8.1 ± 0.41^a^	0.99 ± 0.09^b^	1.97 ± 0.13^a^	**4**.**6 ± 0**.**03** ^**b**^	0.17 ± 0.01^c^	12.44 ± 0.64^a^	**16**.**74 ± 0**.**2** ^**b**^	1.55 ± 0.04^c^	8.9 ± 0.2^a^	9.55 ± 0.23^a^	4.2 ± 0.11^b^

Letters indicate significant differences (p < 0.05) between treatments. Numbers in bold show growth promotion in 0.1 mg ml^−1^ chitosan treatment vs. MS untreated controls. MRL: Maximum root length, RFW: Root fresh weight, SFW: Shoot fresh weight. SA: Salicylic acid, JA: Jasmonic acid.

### Chitosan reduces root growth and development of tomato and barley plantlets

**Figure 7:**
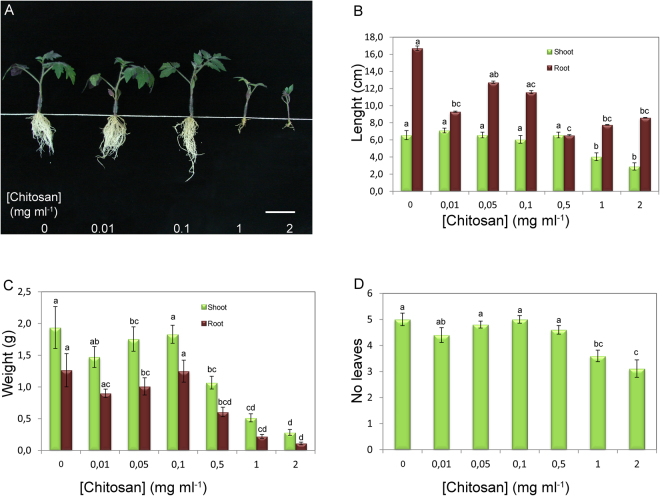
Irrigation with chitosan inhibits tomato root and shoot development. (**A**) Overview of the inhibitory effect of chitosan on development of tomato plantlets after 21 days of treatment. (Scale bar 5 cm). (**B**–**D**) Effect of chitosan on tomato (**B**) shoot and root length, (**C**) biomass of shoot and roots and (**D**) the number of leaves per plant. Different letters indicate significant differences (p < 0.05) between treatments.

To test the effect of chitosan on root development in other plants, we investigated the behaviour of two phylogenetically distant plants from Arabidopsis, tomato (*Solanum lycopersicum* Mill cv. Marglobe; eudicot) and barley (*Hordeum vulgare* L. var. Disticum; monocot). We showed that at concentrations higher than 0.1 mg ml^−1^ chitosan also impaired development of tomato (Figs 7 and S7) and barley plants (Fig. S8). High doses of chitosan (0.5, 1 and 2 mg ml^−1^) applied in the irrigation system for 21d blocked root elongation and caused a strong decrease (more than 2.5-fold) in tomato plant biomass (Fig. 7B and C) and reduced the number of leaves per plant (Fig. 7D). Application of chitosan also induced accumulation of violaceous compounds (likely anthocyanins) on the abaxial side of tomato leaves (Fig. S7). This is perhaps an indication of the stress suffered by plants irrigated with chitosan. However, low doses (0.01–0.1 mg ml^−1^) did not affect tomato plant growth and development.

High chitosan doses also reduced growth of barley plants (Fig. S8). Plants irrigated with 1 and 2 mg ml^−1^ chitosan for 21d showed roots 2-fold shorter than those of controls (Fig. S8A and S8B). However, shoot length remained unaffected. On the contrary, barley plants irrigated with low doses of chitosan (0.01 and 0.1 mg ml^−1^, Fig. S8) exhibited a slight induction of root and shoot growth (Fig. S8B).

### Chitosan blocks cell differentiation and alters root architecture in tomato plants

**Figure 8:**
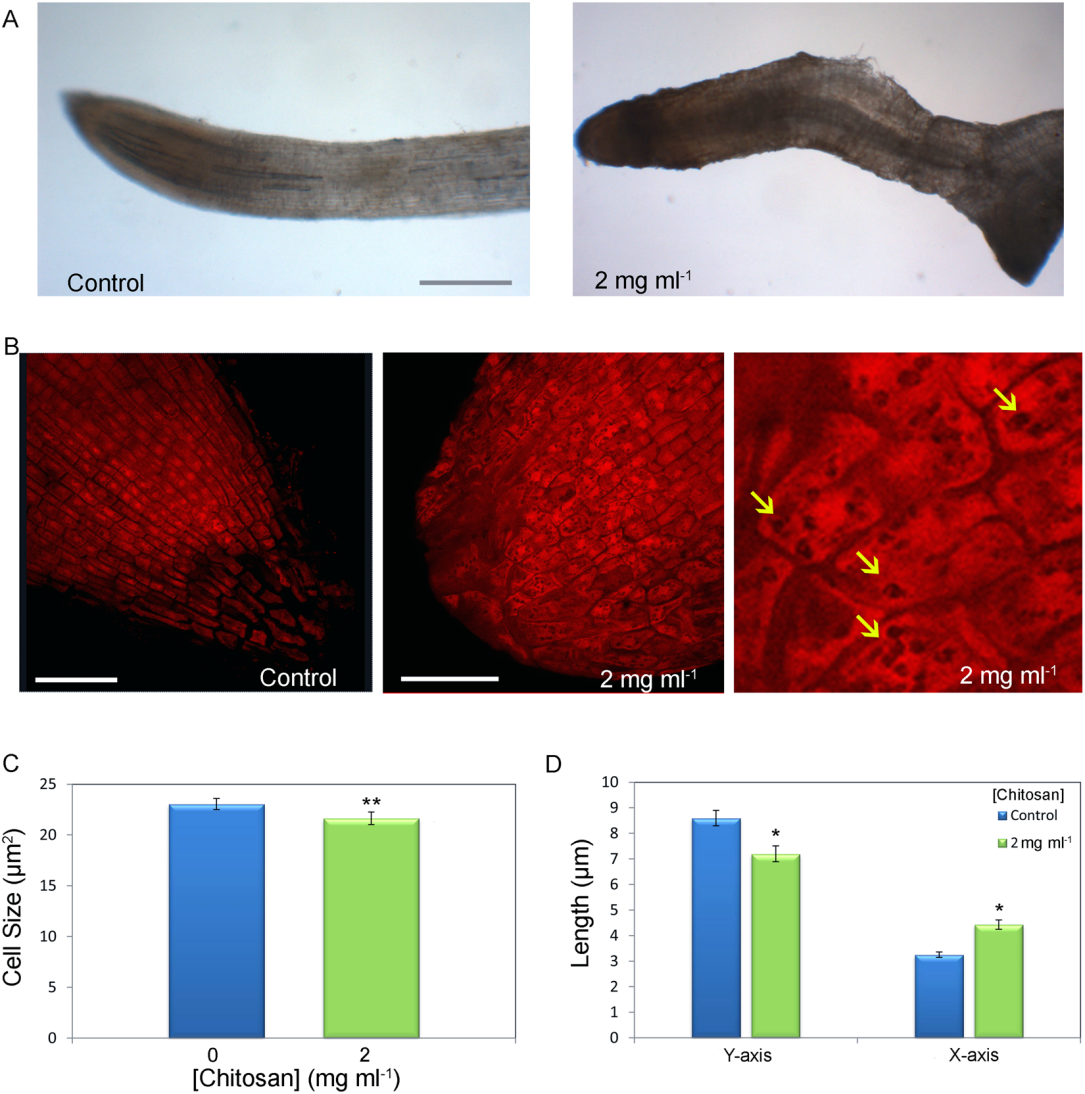
Chitosan affects architecture of tomato roots altering cell division. (**A**) Root apex of plant irrigated with water (control) and chitosan (2 mg ml^−1^) for 21d under visual light. (Scale bar 250 µm). (**B**) Microscopically evaluation of root apices morphology from control plants (left) and plants irrigated with chitosan (centre, right) for 21d. Close-up (right) shows dark intracellular vesicles (arrows) in cells from chitosan treated roots. (Scale bar 50 µm). (**C**) Measurement of tomato root cell size irrigated with chitosan during 21d. (**D**) Effect of chitosan on root cell shape. Cells Y-axis (in the root’s growth direction) and X-axis (perpendicular to growth direction). Asterisks indicate significant differences *(p < 0.05) and **(p < 0.01).

Chitosan changed tomato root development and root cell architecture (Fig. 8). Irrigation with 2 mg ml^−1^ chitosan for 21d altered morphology of tomato roots (Fig. 8A). It caused deformation of the root apex and caused accumulation of dark compounds (like phenols) as described in Arabidopsis, which might be associated with the response to stress. Besides, root growth lost polarity and root apices displayed a round shape, unlike sharp apices from control plants (Fig. 8). Cells from untreated control plants were rectangular and formed strips in the direction of root elongation (Fig. 8B). Instead, root apex cells from chitosan-irrigated plants were isodiametric, and not organised in a given direction (Fig. 8B). Chitosan exposed roots accumulated dark vesicles in their cytoplasm likely related to stress response. Cell imaging analysis showed that chitosan caused a significant reduction (p < 0.01) in the size of root cells (Fig. 8C) as well as changes in their shape, being both shorter and wider (Fig. 8D).

## Discussion

Chitosan has wide applications in agriculture^4^. It has been used as a plant defence inducer^33^ and against infections caused by viruses, bacteria, fungi and nematodes in plants^3^. Most chitosan applications have been performed on the phylloplane as modulator of defences and innate immune responses in plants^34^. However, recent studies have analysed the effect of chitosan applied in the rhizosphere in order to improve plant yield and to enhance virulence of biocontrol fungi such as *P*. *chlamydosporia* ^24,35,36^. In our study we use Arabidopsis to understand the mechanisms underlying the activity of chitosan on root growth. Chitosan causes important changes on Arabidopsis development, reducing root growth and delaying plant development. The effect of chitosan on root development is concentration dependent; 0.01–0.1 mg ml^−1^ chitosan cause small changes on plant growth, while 0.5–2 mg ml^−1^ chitosan arrest Arabidopsis root elongation. Chitosan also reduces the rate of secondary roots emerged in Arabidopsis. Secondary root emergence plays an essential role in water and nutrient uptake^37^. Their inhibition could explain the distortion caused by chitosan on plant development. Besides, chitosan causes accumulation of auxin in Arabidopsis roots. This is due to increased IAA biosynthesis since *YUC2*, *AMI1* and especially *AAO1* are overexpressed in chitosan treated roots. Chitosan also causes auxin accumulation by blocking *PIN1* gene expression involved in IAA transport. Decreases in primary root length and reduction in secondary root emergence caused by chitosan could be explained by auxin overproduction in roots^38,39^.

Auxin accumulation is known to repress the homeodomain transcription factor *WOX5* mediated by *ARF1*, *ARF6* and *ARF10* ^30^. Chitosan causes *WOX5* repression, reducing its localisation in the neighbouring QC cells. *WOX5* is a major regulator of root stem cell activity in the quiescent centre which controls cell division. This could explain why chitosan alters root cell dynamics (root cell shape and size changes) and reduces root elongation. These changes would be directly related to the alteration in the rate of cell division in roots and the morphological changes caused by chitosan. Chitosan also represses *AQC1* gene expression suggesting regulation of cell division in the root meristem. To this respect the effect of chitosan on cell cycle regulators such as cyclins mediated by SHORT-ROOT/SCARECROW pathway, up-stream of *WOX5* ^40^, should be considered in future studies.

The systemic effect of chitosan in plant development could also be explained by changes in homeostasis of hormones other than auxins. Previous authors described chitosan as pathogen/microbe-associated molecular patter (PAMP/MAMP)^41^, which modulates stress-related hormones. In this work we show that chitosan reduces the presence of ABA in roots. This may be related to ABA translocation from root to aerial parts under stress conditions^42^. However, chitosan causes accumulation of JA and SA^43,44^ which is reflected in an induction of biosynthetic and signalling of SA and JA genes by this polymer. This indicates that the increase in these stress-related hormones is translated in their perception. Similar results were observed in plants exposed to abiotic stresses such as heat, salt or drought^45^. *ICS1* and *ICS2* (SA biosynthetic genes) and *NPR1* (SA signalling genes) are overexpressed by chitosan. Induction of *NPR1* and SA signalling could trigger plant immunity mainly PTI (PAMP/MAMP Triggered Immunity)^46^. Previous studies described that increased levels of SA induce synthesis and accumulation of phenols^47^ as we found in chitosan-treated roots. Genes related to JA biosynthesis (*AOC3* and *CYP94B*) and signalling (*MYC2*) were also overexpressed by chitosan. In view of these results, we also confirmed plant perception of chitosan induced stress is reflected in the growth increase of JA insensitive mutants. This effect is chitosan concentration dependent since growth promotion in the mutants was abolished under high chitosan dose. This suggests factors in addition to hormone perception such as ROS levels or phenols accumulation are involved in the activity of chitosan during Arabidopsis root growth. Both, JA and SA hormones have been described in the plant response to stress caused by chitosan when is is applied in leaves^42,44,48^. An induction of stress-related hormones under low doses of chitosan could be associated with a moderate stress which is described that causes an induction of flowering^49 as we observe in our experiments^.

However, the application of chitosan on plant roots and its effect on plant growth and plant defence activation has not been fully investigated. Therefore, we set the present study applying chitosan in the root system of tomato and barley to provide evidences about the activity of chitosan on roots. Chitosan overexposure also arrests development of tomato and barley roots, ultimately altering cell size and shape in root tips. These structural modifications of cells could be related to lignification, cytoplasmic acidification, membrane depolarisation and generation of reactive oxygen species caused by chitosan in plant cells^26^. This phenotype has been also observed when a triazine-base synthetic small compound (rootin) is applied to Arabidopsis roots^50^. We also describe the formation of intracellular dark vesicles in cells of tomato roots treated with chitosan. Their autofluorescence suggests that they maybe phenolic compounds accumulated in response to chitosan-induced cell stress. Similar vesicles have also been previously described in *Mimosa pudica* cells treated with chitosan^51^. High doses of this polymer induce peroxidase and polyphenol oxidases, which in turn generate phenolic compounds^3^ and calose depositions^52^ in plant cells. Chitosan also triggers induction of programed-cell death (PCD) in plants^53^. The involvement of PCD in the inhibitory effect of chitosan on root development should be investigated in future studies. Our work suggests that chitosan, a pathogen/molecular-associated molecular pattern (PAMP/MAMP)^41^, can affect plant development via hormone homeostasis modification. Since plant immunity and growth converge^46^ chitosan could induce both PTI (PAMP triggered immunity) and the hormone changes described in this paper.

Our mechanistic study about the effect of chitosan on plant root development opens up new possibilities to implement this polymer in field applications. Doses, frequency and formulation of chitosan should be adjusted to prevent negative effects on plant development as reported in this study. Besides, we have also proven that chitosan is an experimental tool to manipulate root development, physiology, gene expression and hormone homeostasis. Our study contributes to the understanding of chitosan in the rhizosphere. This will help to develop chitosan application to control important pest and diseases of economically important crops by balancing plant immunity and yield.

## Material and Methods

### Plant material

*Arabidopsis thaliana* (L. Heynh), ecotype Columbia (Col-0) reporter lines, transformants DR5rev:GFP and DR5:GUS^54^, WOX5:GFP^29^, PIN1:PIN1-GFP^55^, YUC2:GUS^56^, mutants *npr1–1* ^57^, *coi1–40* ^58^ and the triple mutant *myc2/3/4* ^59^, were tested with chitosan. Arabidopsis seeds were surface-sterilised using 1% NaClO for 2 min and then washed 3 times with sterile distilled water. Surface sterilised seed were stratified at 4 °C for 48 h and then grown on MS (Murashige and Skoog medium; Sigma) plates as in Ripoll *et al*.^60^.

Tomato (*Solanum lycopersicum* Mill cv. Marglobe) and barley (*Hordeum vulgare* L. var. Disticum) seeds were also surface-sterilised using 50 ml 4% sodium hypochlorite with 3 drops of Tween-20 (Sigma-Aldrich) for 20 min and 1 h, respectively. They were then washed three times (5 min each) with sterile distilled water. Surface-sterilised seeds were plated on 9 cm petri dishes with a germinating medium (1.2% agar supplemented with glucose (10 g l^−1^), peptone (0.1 g l^−1^) and yeast extract (0.1 g l^−1^)^61^. Seeds were stratified for two days at 4 °C and then incubated at 25 °C in the dark for 5 days and finally 4 days under a 16 h/8 h (light/darkness) photoperiod.

### Chitosan

Medium molecular weight chitosan (T8: 70 kDa and 85% deacetylation degree) was from Marine BioProducts GmbH (Bremerhaven, Germany). Chitosan was prepared as described in Palma-Guerrero *et al*.^62^. The resulting solution was dialyzed against distilled water and then autoclaved. It was stored at 4 °C until used and never kept longer than 5 weeks.

### Characterisation of Arabidopsis growth and development with chitosan in solid media

Arabidopsis seeds (Col-0, DR5rev:GFP, DR5:GUS, WOX5:GFP, YUC2:GUS, PIN1:PIN1-GFP, c*oi1–40*, *myc2/3/4* and *npr1–1*) were surface sterilised. Seeds were then plated on MS medium (Sigma) supplemented with 0.05% 2-(N-morpholino) ethanesulfonic acid (MES, Sigma), 1% sucrose and 1% technical agar and amended with chitosan. Plates with no chitosan were used as controls. Seed were placed at 4 °C for 48 h in the dark to synchronise germination. They were then incubated at 21 °C and 65% relative humidity (RH) with continuous light for 21d upright to allow root elongation. Every two days plants were checked for secondary root and bolting emergence in order to identify flowering time. At harvest time, total plant weight, root weight and length and number of leaves in the rosette per plant and treatment were scored. This experiment was performed in triplicate.

Intracellular hydroxyl, peroxyl and other reactive oxygen species were detected using 2′-7′ dichlorodihydrofluorescein diacetate (H2DCFDA; Sigma, St. Louis, MO, USA). Arabidopsis roots were exposed to 1 mg ml^−1^ chitosan for 2 h and then were dipped in H2DCFDA. Finally, we observed ROS in roots by confocal microscopy with a 490 nm excitation and a 535 nm emission filters. Untreated control roots were incorporated to evaluate the ROS levels in standard conditions.

### Characterisation of the physiological and cellular effect of chitosan on Arabidopsis roots

Five-day-old Arabidopsis Col-0, DR5rev:GFP, PIN1:PIN1-GFP and WOX5:GFP seedlings obtained as above were transferred to liquid MS medium amended with 0.1 and 1 mg ml^−1^ chitosan. Liquid MS without chitosan was used as control. After 0–150 min (primary roots) or 24–72 h (secondary roots) in contact with chitosan, seedlings were sampled to determine root reaction to chitosan. Roots were stained with a drop 50 µg ml^−1^ propidium iodide (PI) for root cell wall labelling. PI and GFP fluorescence were detected in root cells using respectively 488-nm and 580–620 nm (autof.) or 505–530 nm (GFP) as excitation and detection wavelengths in the laser confocal microscope. GFP expression in micrographs was quantified using MetaMorph (Molecular devices, Sunnyvale, Ca, USA). These experiments were performed per triplicate.

### Plant hormone quantification in Arabidopsis roots treated with chitosan

Arabidopsis Col-0 wild-type plants were grown under sterile conditions for 4d on upright plates containing 0.5x MS agar. Plants were then transferred to plates with MS (controls) or MS amended with 1 mg ml^−1^ chitosan and incubated upright for 3d. Roots from ca. 50 plants per treatment were collected and ground in liquid nitrogen. Approximately 100 mg of ground tissue (n = 4 per treatment) per sample and treatment (control/chitosan) were used for hormone extraction. Root tissue was suspended in 80% methanol-1% acetic acid containing internal standards and mixed by 1 h shaking at 4 °C. The extract was centrifuged at −20 °C and the supernatant dried in a vacuum evaporator overnight. Dry residue was dissolved in 1% acetic acid and passed through an Oasis HLB (reverse phase) column as described in Seo *et al*.^63^. For gibberellic acid (GAs), indole acetic acid (IAA), abscisic acid (ABA), salicylic acid (SA) and jasmonate (JA) quantification, dried eluate was dissolved in 5% acetonitrile and 1% acetic acid. Hormones were separated using reverse phase UHPLC chromatography (2.6 µm Accucore RP-MS column, 50 mm length × 2.1 mm i.d.; ThermoFisher Scientific) with a 5 to 50% acetonitrile gradient containing 0.05% acetic acid, at 400 µL/min for 14 min. Internal standards for quantification were deuterium-labelled hormones except for JA where dhJA was used. Hormones were identified with a Q-Exactive mass spectrometer (Orbitrap detector; ThermoFisher Scientific) targeted Selected Ion Monitoring (SIM). Hormone concentrations in the extracts were determined using embedded calibration curves as well as program Xcalibur 2.2 SP1 build 48 and TraceFinder.

### Hormone Treatments

To evaluate the relevance of hormone balance in the response of Arabidopsis to chitosan, we tested the importance of IAA, SA and JA including these hormones in the MS media. After IAA, SA and JA quantification in Arabidopsis roots, we prepared MS medium amended with these hormones alone and in combination in the same amount (+10%) quantified in Arabidopsis roots exposed to chitosan (Table 1). We also included MS medium (negative control) and MS amended with 1 mg ml^−1^ chitosan (positive control). Arabidopsis (Col-0) seeds were then plated on MS solid media, stratified, grown and plant scored as described above for 15d.

### Histochemical Analysis

For histochemical analysis β-glucuronidase (GUS) activity, DR5:GUS and YUC2:GUS^64^ *A*. *thaliana* seedlings (15 day-old) were incubated for 24 h in MS plates amended with 1 mg ml^−1^ chitosan. MS plates without chitosan were used for controls. Roots were then incubated overnight at 37 °C in a GUS reaction buffer (0.5 mg ml^−1^ 5-bromo-4-chloro-indolyl-β-D-glucoronide in 100 ml sodium phosphate, pH = 7). Stained seedlings were rinsed in sodium phosphate for 5 min, and then analysed by microscopy^65^. For each marker line, at least 5–7 transgenic plants were analysed. A representative plant was chosen and photographed, using a digital camera connected to a binocular microscope Nikon SMZ1500 and analysed using ACT-1 2.70 software.

### Quantification of gene expression by qRT-PCR

RNA was obtained from eighty roots of 15day-old Col-0 plants exposed for 24 h to chitosan (0.1 or 1 mg ml^−1^) in MS plates. MS plates without chitosan were used as controls. Roots were frozen and ground and then RNA isolated using Trizol reagent (Thermo Fisher Scientific) following manufacturer’s instructions. Isolated RNA was treated with TurboDNA free (Ambion) for removing DNA remains. cDNA was then synthetized with a retro-transcriptase RevertAid (Thermo Fisher Scientific) using oligo dT (Thermo Fisher Scientific). Gene expression was quantified using real-time reverse transcription PCR (qRT-PCR). SYBR Green with ROX (Roche) was used following the manufacturer instructions. Gene quantifications were performed in a Step One Plus real-time PCR system (Applied Biosystems). Relative gene expression was estimated with the ∆∆Ct methodology^66^ with three technical replicates per condition. Primers used to quantify the expression of genes related to Arabidopsis response to chitosan are shown in Table S1. *ACTIN2* (*ACT2*) and *ORNITHINE CARBAMOYLTRANSFERASE* (*OTC*) genes were used as endogenous controls for all experiments, since their expression showed Ct stability for all conditions tested.

### Application of chitosan to the irrigation system of tomato and barley plantlets

Tomato and barley seedlings were planted individually in 150 ml sterile cylindrical containers (Deltalab) each containing 70 cm^3^ sterilised sand. Twenty-tree ml of 1/10 Gamborg’s B-5 basal medium (Sigma) amended with chitosan (0.01; 0.05; 0.1; 0.5; 1 and 2 mg ml^−1^) were used to irrigate each seedling. Seedlings watered with 1/10 Gamborg’s only were used as controls. Seedlings were incubated at 25 °C, 65% relative humidity and under a photoperiod (16 h light/8 h dark) in a growth chamber (Fitoclima 10000EHVP). Twenty-one days after planting, 10 plants per treatment were sampled. Fresh shoot weights (FSW), maximum shoot length (MSL), fresh root weight (FRW) and maximum root length (MRL) were measured per plant^67^. For tomato plants, the number of leaves per plant was also scored. These experiments were replicated 4 times.

### Evaluation of cell morphology in tomato plants exposed to chitosan

To assess the effect of chitosan on tomato roots we evaluated root apex cell morphology. The primary root apex from 20 day-old plants was observed in a Leica TCS-SP2 laser-scanning confocal microscope using 488-nm and 580–620 nm as excitation and emission wavelengths^68^. We scored at least 75 cells per root apex from 3 plants both irrigated with chitosan and controls. We measured Y-axis (root growth direction), X-axis (perpendicular to Y-axis) and surface for each root cell in a 200 × 200 pixels area using Image Analysis Software MetaMorph.

### Statistical analyses

Analysis of variance (ANOVA) was used to test for significant differences in experiments^69^. Homogeneity of variance was checked using Bartlett’s test^70^. When significant heterogeneity was found, data were 1/x transformed and then ANOVA F-test (p-value < 0.05) was used. When significant heterogeneity was not removed by transformations, analyses were performed on the untransformed data, but F-test α-value was set at 0.01^69^. Post-hoc analyses were conducted using Tukey HSD comparisons. Analyses were performed using R version 3.0.2. (The R Foundation for Statistical Computing, 2015). All data were reported as mean ± standard error (SE) and statistical tests were conducted with significance level α = 0.05.

## Electronic supplementary material

Supplementary Material
