# Supplementary material for: Induction of auxin biosynthesis and WOX5 repression mediate changes in root development in Arabidopsis exposed to chitosan
Source: Sci Rep. 2017 Dec 1;7:16813. doi: 10.1038/s41598-017-16874-5 (PMC5711845; doi:10.1038/s41598-017-16874-5)
Supplement: Supplementary file 1 — Supplementary Material [file 41598_2017_16874_MOESM1_ESM.pdf]

Article title: **Induction of auxin biosynthesis and *WOX5* repression mediate changes in root development in Arabidopsis exposed to chitosan**

Authors: Federico Lopez-Moya[1*], Nuria Escudero[1,2], Ernesto A. Zavala-Gonzalez[1,3], David Esteve-Bruna[4], Miguel A. Blázquez[4], David Alabadí[4] and Luis V. Lopez-Llorca[1]

[1] Laboratory of Plant Pathology, Multidisciplinary Institute for Environment Studies (MIES) Ramón Margalef. Department of Marine Sciences and Applied Biology. University of Alicante, Alicante, Spain.[2] Departament d'Enginyeria Agroalimentària i Biotecnologia, Universitat Politècnica de Catalunya, Barcelona, Spain.[3] Atlántica Agrícola Company SA. Villena, Alicante, Spain.[4] Instituto de Biología Molecular y Celular de Plantas, Consejo Superior de Investigaciones Científicas-Universidad Politécnica de Valencia, Valencia, Spain.

**Figure S1. Chitosan induces reactive oxygen species accumulation in Arabidopsis roots.**

**Figure S2. Chitosan provokes the accumulation autofluorescent compounds in Arabidopsis primary roots.**

**Figure S3. Chitosan reduces expression of WOX5*:*GFP in secondary roots of Arabidopsis**.

**Figure S4. Chitosan induces *AQC1* gene expression at low chitosan doses.**

**Figure S5. Chitosan induces expression of genes involved in SA and JA biosynthesis.**

**Figure S6. Reduction of Arabidopsis root length by combined exogenous application of hormones induced by chitosan (IAA+SA+JA).**

**Figure S7. Chitosan induces accumulation of violaceous compounds in the abaxial side of tomato leaves**.

**Figure S8. Chitosan irrigation arrests growth of barley roots.**

**Table S1. Primers used to quantify expression of genes related with auxin metabolism using qRT-PCR.**

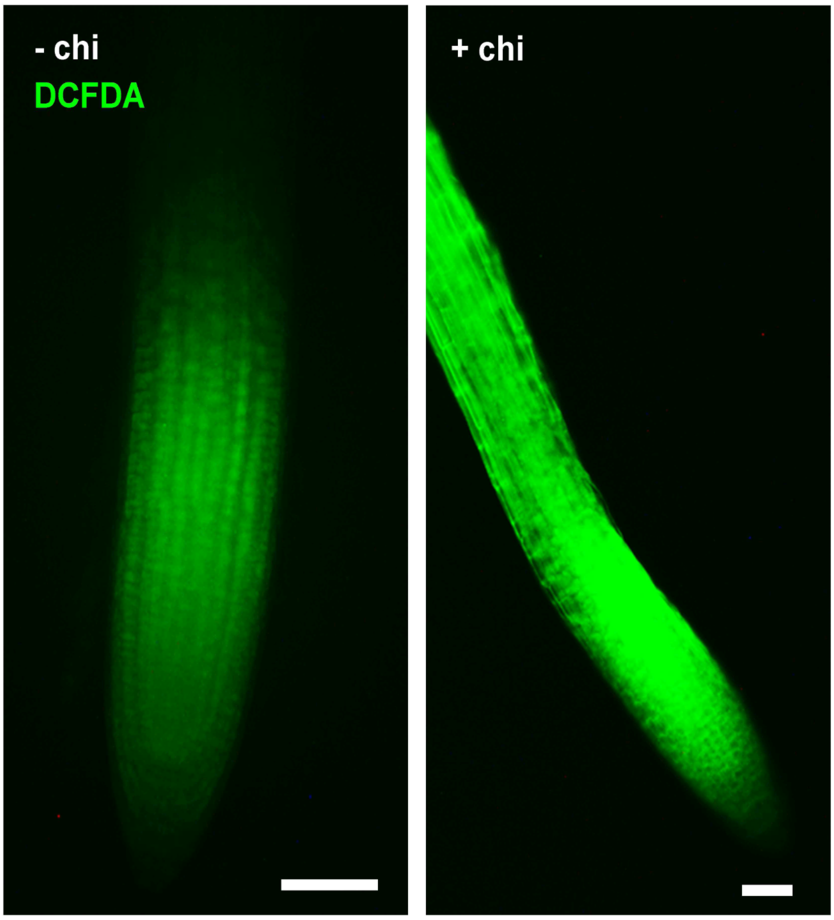


FigureS1

**Figure S1.** Chitosan induces reactive oxygen species accumulation in Arabidopsis roots. Exposure of Arabidopsis plants to chitosan induces accumulation of ROS in roots measured by H2DCFDA staining. (Scale bar 25 µm).

FigureS2

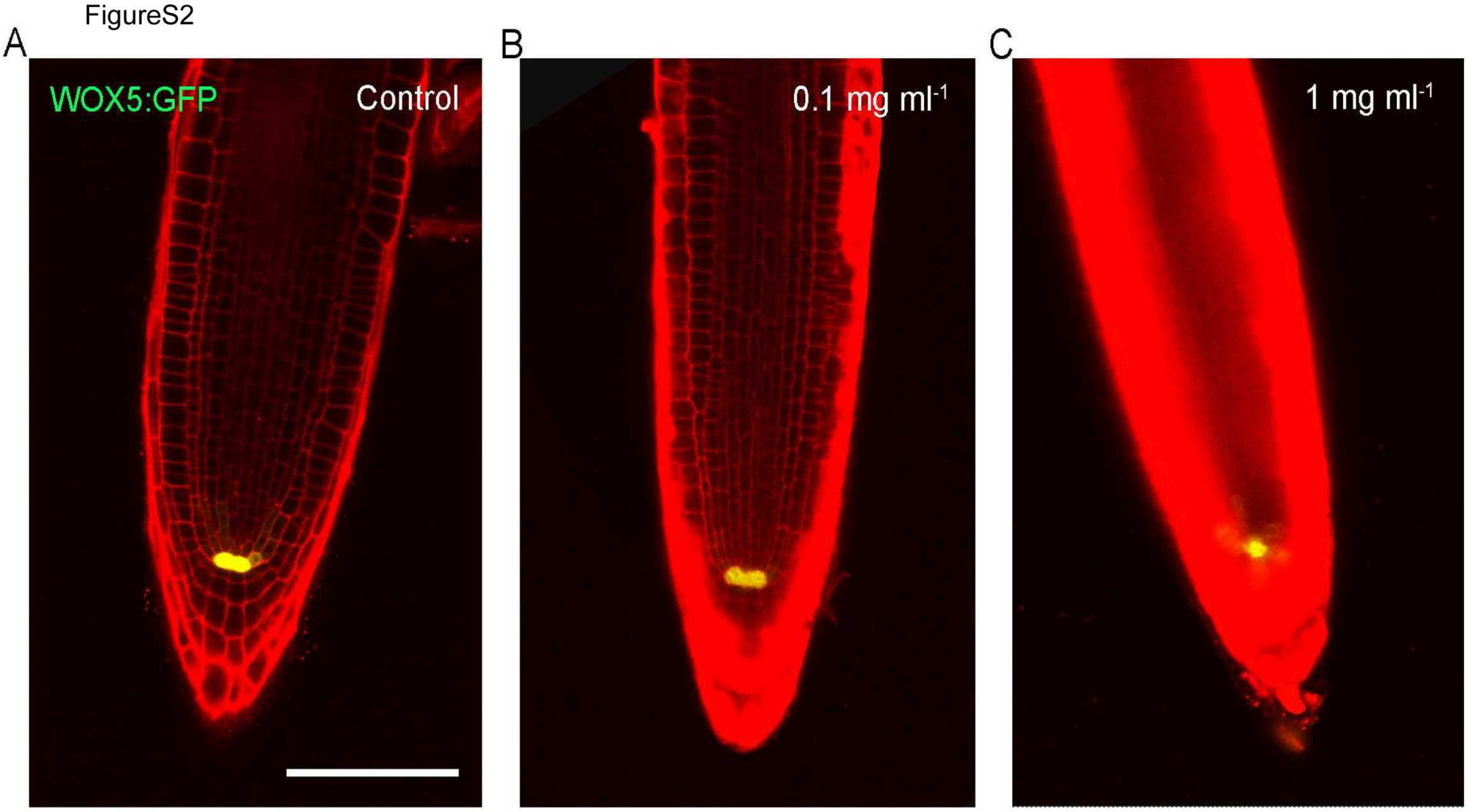


**Figure S2.** Chitosan provokes the accumulation autofluorescent compounds in Arabidopsis primary roots. Autofluorescent zones are usually associated with deposition of phenolic and calose depositions. Green fluorescence indicates WOX5:GFP expression in roots exposed to different chitosan concentrations. (Scale bar 50 µm).

FigureS3

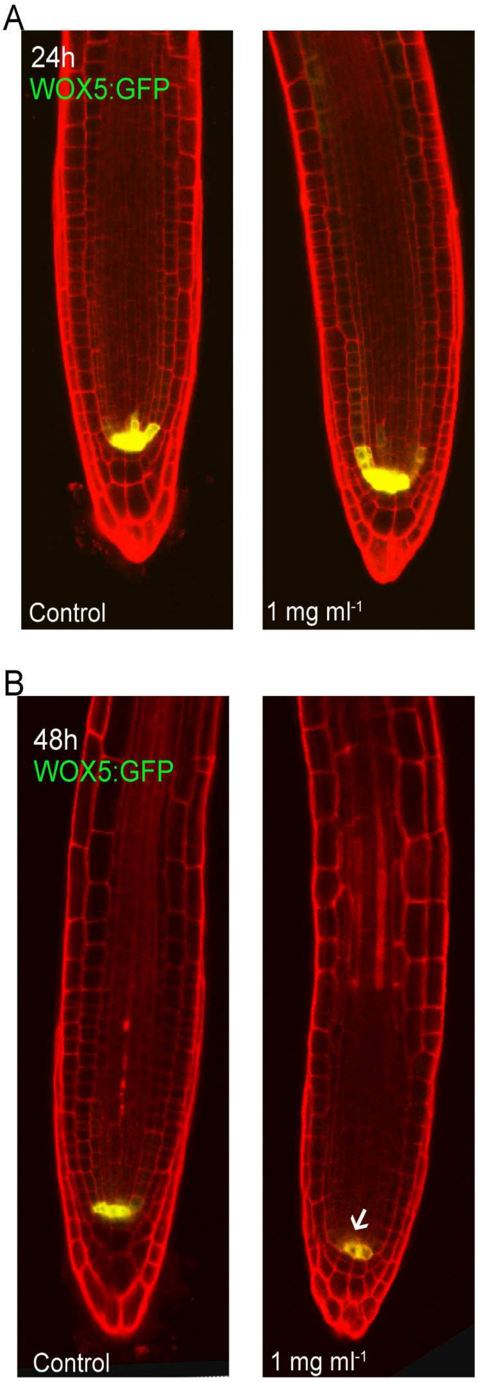


**Figure S3.** Chitosan reduces expression of WOX5:GFP in secondary roots of Arabidopsis. WOX5:GFP expression at 24h (A) and 48h (B). Arrows show WOX5:GFP repression in plants treated with chitosan in comparison with untreated controls.

FigureS4

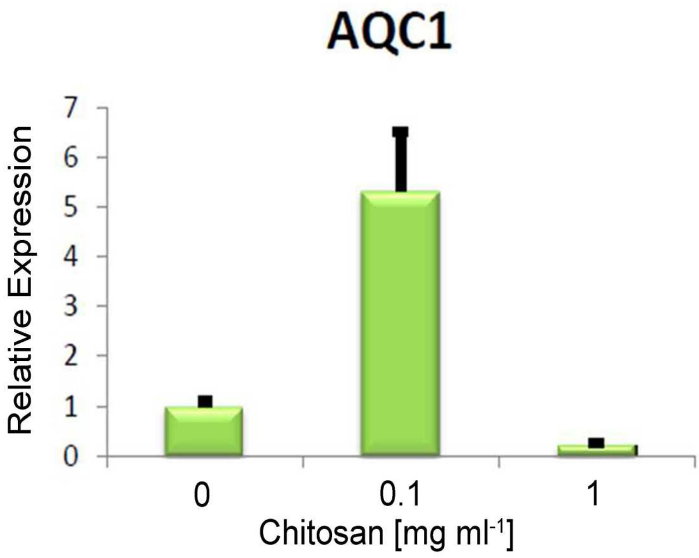


**Figure S4**. Chitosan induces *AQC1* gene expression at low chitosan doses. Low chitosan doses (0.1 mg $ml^{-1}$) propitiate an overexpression of *AQC1* gene related to cell division regulation in the root meristem. High doses of chitosan (1 mg $ml^{-1}$) repress gene expression of those genes affecting cell division and reducing root elongation.

Figure S5

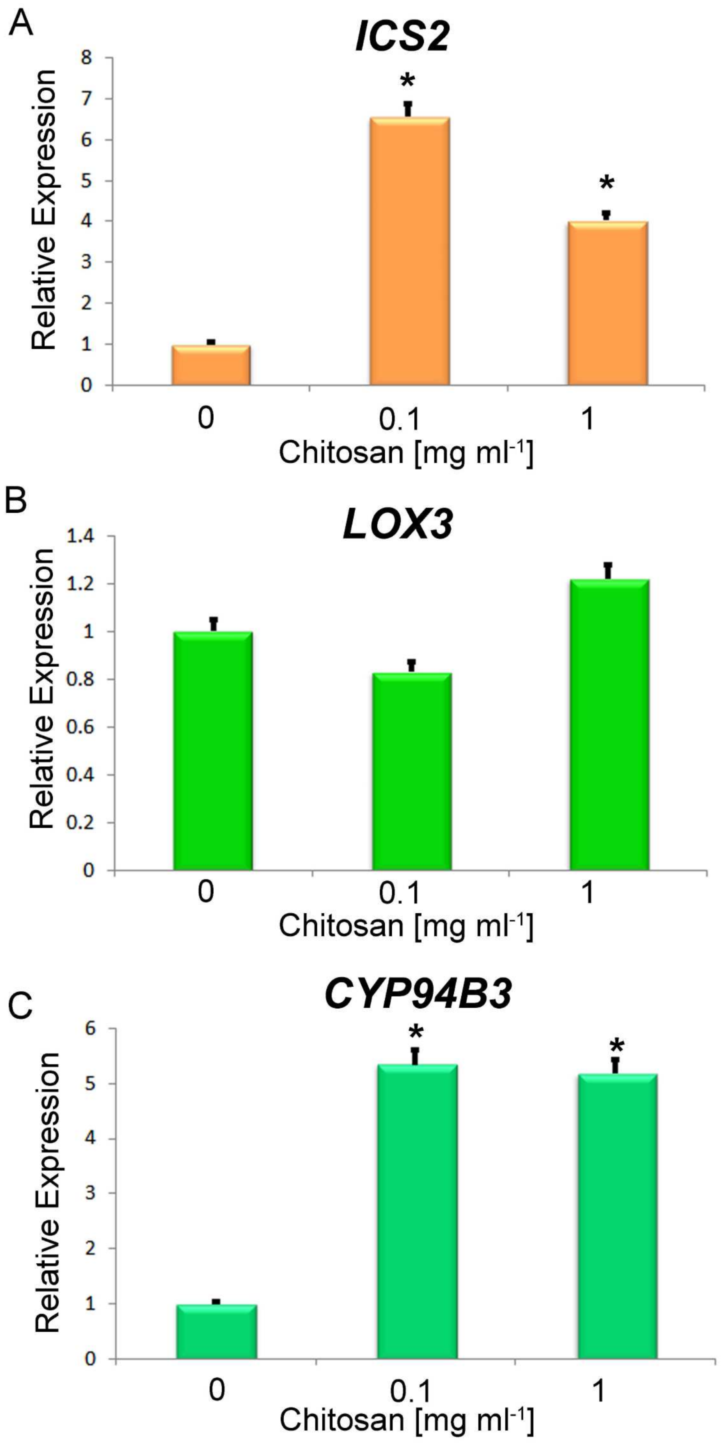


**Figure S5**. Chitosan induces expression of genes involved in SA and JA biosynthesis. **A)** *ICS2* (SA biosynthesis), **B)** *LOX3* (JA biosynthesis) and **C)** *CYP94B3* (JA biosynthesis).

Figure S6

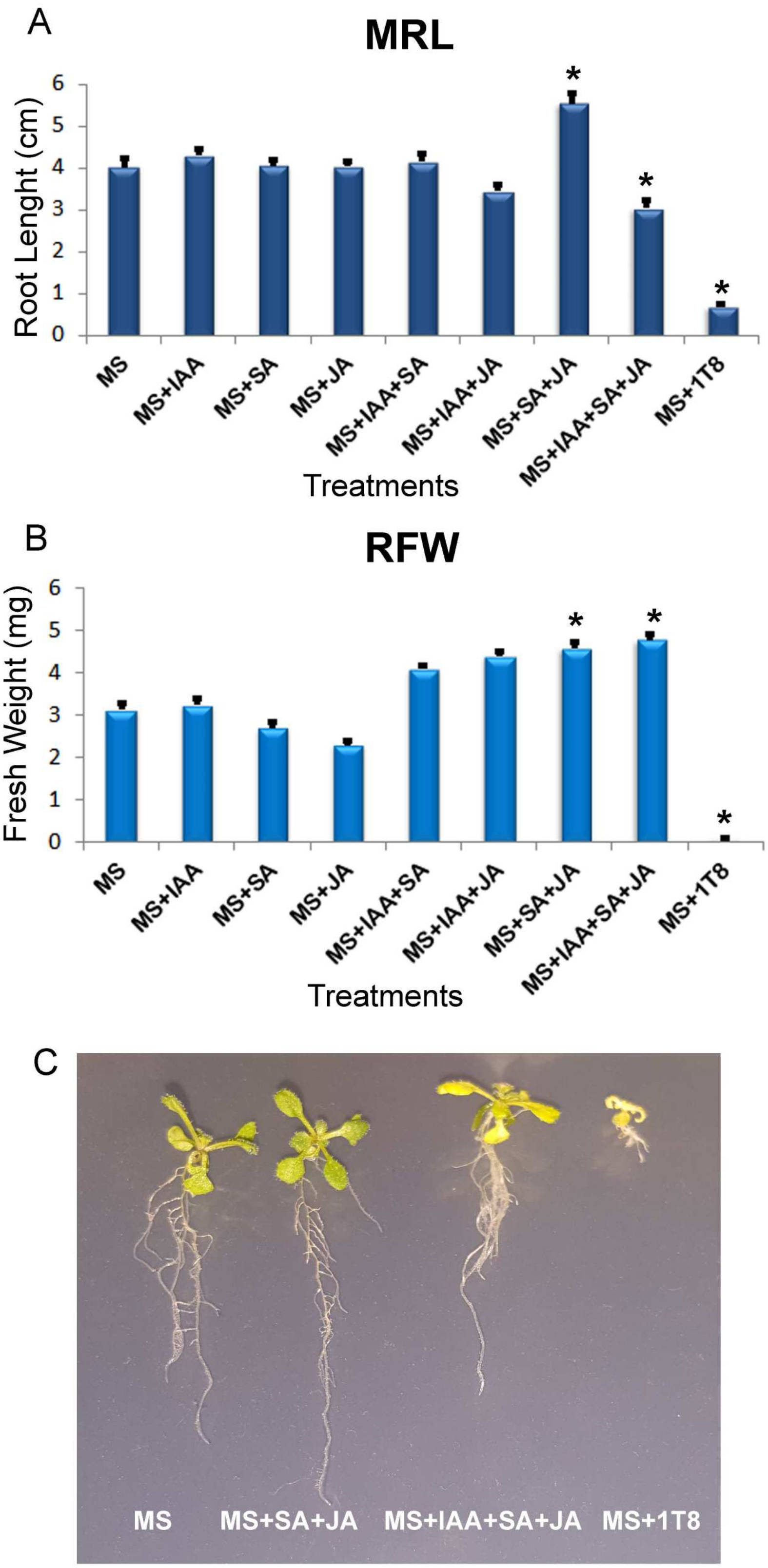


**Figure S6.** Reduction of Arabidopsis root length by combined exogenous application of hormones induced by chitosan (IAA+SA+JA). **A)** Maximum root length (MRL). **B)** Fresh root weight (FRW). **C)** Example of plants grown on MS medium amended with hormones combinations and controls. Hormones were amended to MS at the same amount (+10%) quantified in plants exposed by chitosan (Table 1). Abbreviations: MS: Murashige and Skoog medium; IAA: indole acetic acid; SA: salicylic acid; JA: jasmonic acid. 1T8: 1 mg $ml^{-1}$ chitosan.

Figure S7

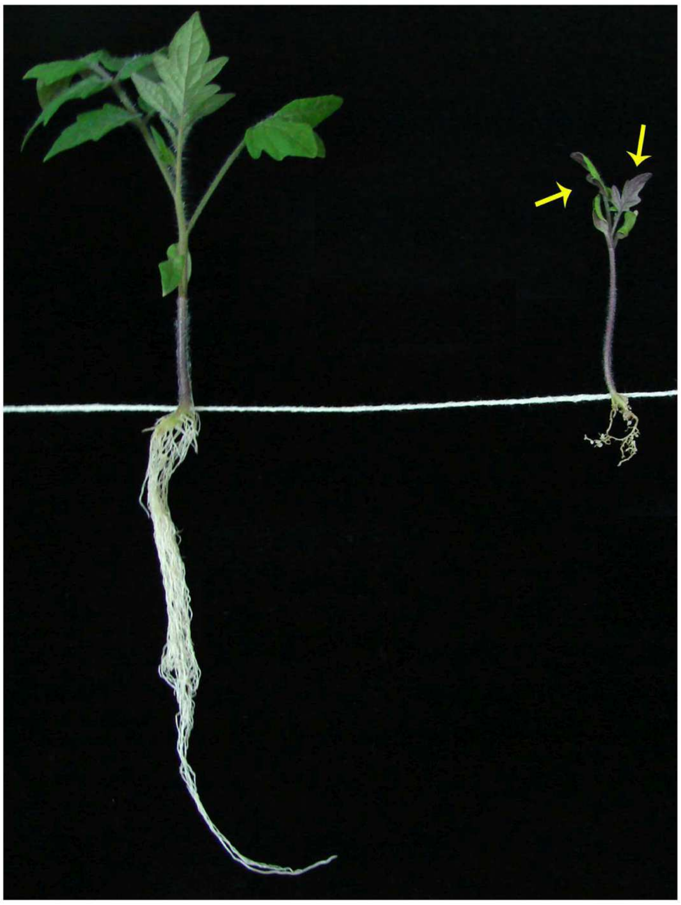

**Figure S7.** Chitosan induces accumulation of violaceous compounds in the abaxial side of tomato leaves. Yellow arrows indicate accumulation of anthocyanins related to the response of tomato plants to stress when they were irrigated with chitosan (2 mg $ml^{-1}$) for 20d. Root and shoot growth were arrested by chitosan in comparison with untreated controls.

Figure S8

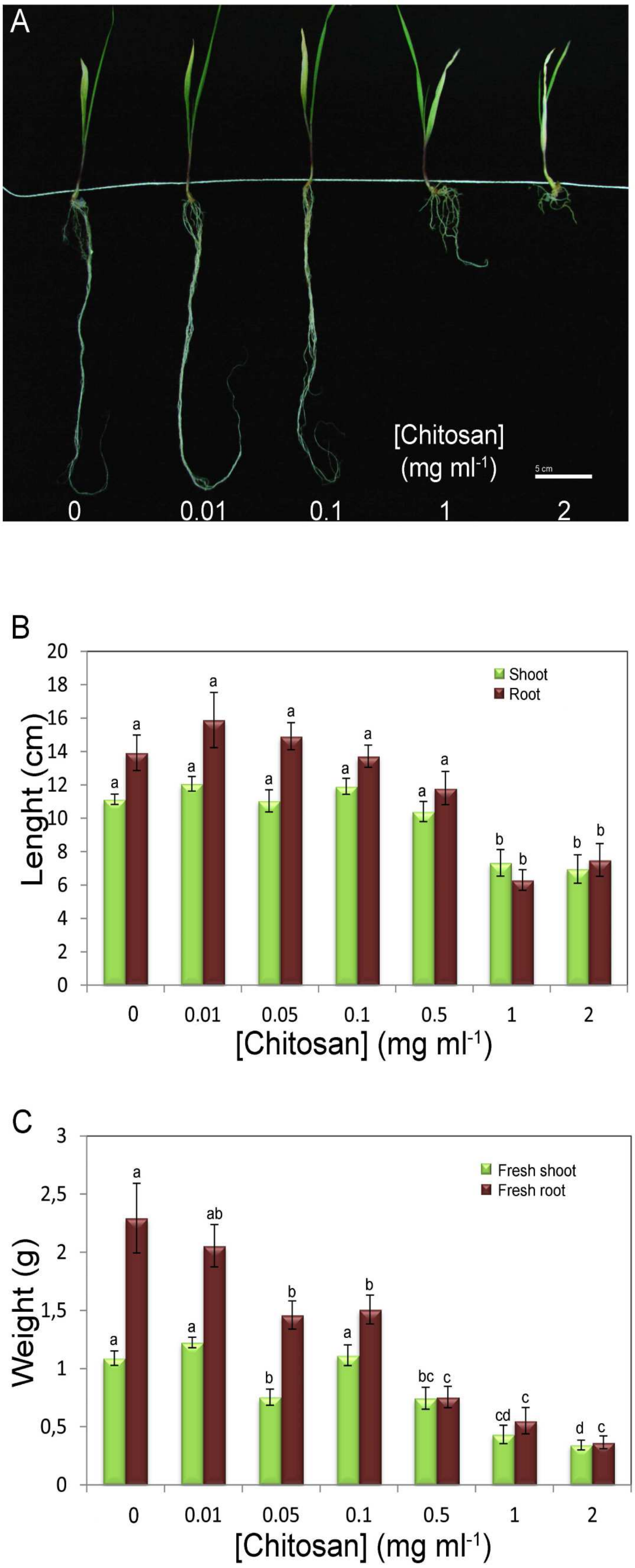


**Figure S8.** Chitosan irrigation arrests growth of barley roots. **A)** Overview of the inhibitory effect of chitosan on development of barley plantlets after 21d treatment. (Scale bar 5 cm). **B)** Effect of chitosan on barley root and shoot development. **C)** Effect of chitosan on fresh biomass of barley shoot and root. Different letters indicate significant differences ($p<0.05$) between treatments.

**Table S1.** Primers used for quantification hormone-related gene expression by qRT-PCR

| **Primer Name** | **5'→3'** |
|---|---|
| HK_ACT2_fw | TCCCTCAGCACATTCCAGCAGAT |
| HK_ACT2_rev | AACGATTCCTGGACCTGCCTCATC |
| HK_OTC_fw | TGAAGGGACAAAGGTTGTGTATGTT |
| HK_OTC_rev | CGCAGACAAAGTGGAATGGA |
| WOX5_fw | CTATTGGTTTCAGAATCATAAGGCTA |
| WOX5_rev | TGACAATCTTCTTCGCTTATTTCA |
| AQC1_fw | GGAGCGACTTTTCAGATTGC |
| AQC1_rev | ATTGGACGGAACCACTTGAG |
| AMI1_IAM_fw | CCAGGTCCTCCACCGCAT C |
| AMI1_IAM_rev | TTCGTGTAGCCCCAATGGTATG |
| PIN1_fw | ATCTCCGAGCAGTTTCCAGA |
| PIN1_rev | CGCAGATAAGCCTTGAGACC |
| YUC2_fw | TAGCGTCACTATGGCAGCAC |
| YUC2_rev | CCTCCACGGTCTGGTTAAAA |
| AAO1_fw | AGAGCGTCAAGCTTGGTTGT |
| AAO1_rev | GCATTGTGTGGCATGAAAAC |
| ARF1_fw | TGAATGGCATTTTAGGCACA |
| ARF1_rev | ATGTGCCGCCTAACACCTAC |
| ICS2F1 | ATTGGCAGGACAGCTAAAAAGAG |
| ICS2R1 | AGCTGACTCATGTTCAAGTGACTT |
| ICS1F | AGTGAATTTGCAGTCGGGATCAG |
| ICS1R | TCGCCTGTAGAGATGTTGTTGC |
| NPR1F | GGACACAAGAAGAAGGCCAAGA |
| NPR1R | TCCATCACCGTTGACTTCAACATC |
| AOC3-1F | CGGTCCAGATTTCTCCTCCCGAT |
| AOC3-1R | CACCGCTCTTTTCAGGAACGTGT |
| CYP9-1F | GCCATGAGGCTATATCCTCCAGTT |
| CYP9-1R | CCGGCACAATCTCAAACCGACTCA |
| LOX3-1F | TCGCACGTCAAGCCATAGCTGGA |
| LOX3-1R | GCGCTCAATTGCCTGTGTGCAGCT |
| MYC2-S | CAAGGAGGAGTGTTTGGGATGC |
| MYC2-AS | GTCGAAAAATTAAGTTCTCGGGAG |

HK: indicates primers used to amplify genes used as a housekeeping
